# Tuning the isoelectric point of graphene by electrochemical functionalization

**DOI:** 10.1038/srep11794

**Published:** 2015-07-02

**Authors:** Laura Zuccaro, Janina Krieg, Alessandro Desideri, Klaus Kern, Kannan Balasubramanian

**Affiliations:** 1 Max Planck Institute for Solid State Research, D-70569 Stuttgart, Germany; 2 Dept. of Biology, University of Rome Tor Vergata, I-00133 Rome, Italy; 3 Institut de Physique de la Matière Condensée, Ecole Polytechnique Fédérale de Lausanne, CH-1015 Lausanne, Switzerland

## Abstract

The ability to control the charge-potential landscape at solid-liquid interfaces is pivotal to engineer novel devices for applications in sensing, catalysis and energy conversion. The isoelectric point (pI)/point of zero charge (pzc) of graphene plays a key role in a number of physico-chemical phenomena occurring at the graphene-liquid interface. Supported by theory, we present here a methodology to identify the pI/pzc of (functionalized) graphene, which also allows for estimating the nature and extent of ion adsorption. The pI of bare graphene (as-prepared, chemical vapor deposition (CVD)-grown) is found to be less than 3.3, which we can continuously modify up to 7.5 by non-covalent electrochemical attachment of aromatic amino groups, preserving the favorable electronic properties of graphene throughout. Modelling all the observed results with detailed theory, we also show that specific adsorption of ions and the substrate play only an ancillary role in our capability to tune the pI of graphene.

Miniaturized devices with nanostructures as active elements are highly promising to realize the dream of complete chemical analysis on a single chip^1^. Among the various candidates graphene as an active element shows high promise due to the very high chemical stability in spite of being just a monolayer of carbon atoms^2^,^3^. Due to the highly correlated 2D electron system, graphene is electronically quite robust and provides for very low electrical resistances and low noise^4^,^5^. Moreover, the vast area of carbon-based chemistry can be directly exploited to engineer the graphene surface for a desired application^6^,^7^,^8^. Unlike in graphite, charge carriers in graphene can be modulated by using an external electric field^9^,^10^, which can be realized in liquid using an electrochemical gate provided by a reference electrode^11^,^12^. This construct, which is quite similar to an ion-selective field-effect transistor (ISFET) configuration^13^, allows for the label-free detection of various chemical and biological species directly in liquids as the binding or reaction takes place^5^,^14^,^15^.

One of the fundamental challenges in such a scenario is the need to have control over the charge-potential landscape of the graphene-liquid interface in addition to providing for the right chemical functionalities and biological receptors for the application of interest. The charge density on graphene has been found to be largely dependent on the nature and density of chemical groups available on the surface^16^. For example, the various preparations steps during growth or during the fabrication of devices may introduce chemical groups (such as oxygen-containing functionalities) that may affect the charge density on the graphene surface. Similar observations have also been made on carbon nanotubes (CNTs)^17^,^18^. The isoelectric point (IEP or pI)/point-of-zero charge (pzc) provides a measure of the acid-base properties of the ionizable groups and in turn the surface charge behavior as a function of solution pH^19^,^20^. At a pH above this value, the surface charge is mainly negative, while it is positive otherwise. Analyte adsorption and charge transfer are interfacial processes that are very sensitive to the surface charge at the graphene-liquid interface and the capability to tune the isoelectric point is hence fundamentally important for the aforementioned applications^21^. On graphene, there is no real estimate of the value of pI/pzc yet. Here, we present a theoretical model and an experimental strategy to determine the isoelectric point of graphene. We arrive at this value using the electrochemical field-effect setup in liquids and measuring the transistor response in a range of solutions with differing pH and ionic strength (IS). Moreover, we show that we can regulate the surface charge distribution by modulating the pI of graphene. To achieve this, we decorate the graphene surface with a variable density of aromatic amino groups by electropolymerization. Chemical functionalization of graphene is widely used to attain new chemical and physical properties that are not attainable on bare graphene^6^,^7^,^8^.

## Results and Discussion

### The graphene-liquid interface

**Figure 1:**
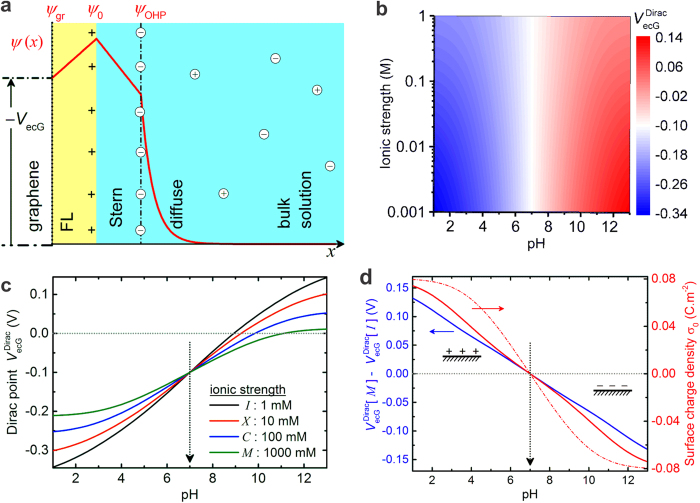
Charge-potential relationships simulated at the graphene-liquid interface. (**a**) The model of graphene-liquid interface utilized here showing the functional layer (FL) containing the functional groups on the graphene surface and the Stern and diffuse layers. The potential profile (*ψ*(*x*)) in the presence of an applied voltage (*V*_ecG_, electrochemical gate voltage) is superimposed, with *ψ*_gr_, *ψ*_0_ and *ψ*_OHP_ referring to the potential at the graphene plane, at the (functionalized) graphene-liquid interface and at the outer Helmholtz plane (OHP) respectively. (**b**) 2D map of gate voltage at the Dirac point (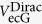) as a function of pH and ionic strength calculated for *ψ*_off_ = −0.1*V*. (**c**) Dirac point profiles (extracted from the 2D-map) as a function of pH for 4 different ionic strength values (*I* : 1 mM, *X* : 10 mM, *C* : 100 mM, *M* : 1 M). (**d**) (blue curve) Difference Dirac curves obtained by subtracting the curve at 1 mM IS from that at 1 M IS (referred to as *M* − *I*). (red curves) Net surface charge density (*σ*_0_) as a function of pH at the two ionic strengths (1 mM – solid line and 1 M – dashed line). It is apparent that the zero crossing of the difference Dirac curve occurs at the point where the charge is zero on the surface corresponding to the assumed isoelectric point (IEP or pI : 7) of the surface. Refer to supplementary information for model parameters. (see also Fig. S1).

First we present a theoretical formulation of the charge-potential relationships at the graphene-liquid interface as shown in Fig. 1(a). We bring together three different models that have been reported for CNTs^17^, graphene^16^ and for the silicon oxide-liquid interface^22^,^23^ within the context of ISFETs. Accordingly, the electrical double layer (EDL) at the interface comprises of the diffuse ionic layer and the Stern layer connected by the outer Helmholtz plane (OHP), which determines the nearest approach of ions from the solution^24^. The charge distribution at the OHP (*σ*_OHP_) can be modelled using the Gouy-Chapman-Stern theory as


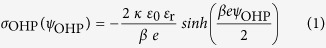


where *ψ*_OHP_ is the potential at the OHP, *β* = 1/*k*T, *κ*^−1^ is the Debye screening length given by 
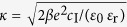
, *c*_I_ is the total ionic strength of the solution, *ε*_0_ and *ε*_r_ are permittivity of free space and relative permittivity and *e* is the electronic charge. On the graphene side, we model the presence of functionalities using a layer of a definite thickness (*t*_FL_) with a dielectric constant (*ε*_r*−*FL_) different from that of water (*ε*_r−H2O_ = 80). The functionalities present ionizable groups on the graphene surface, whose charge distribution can be modeled by a Langmuir-Freundlich type of an isotherm^16^,^17^,^25^ depending on the pH and ionic strength of the solution. Here the acid/base dissociation constants (*K*_a_/*K*_b_ or p*K*_a_/p*K*_b_) of the ionizable groups dictate the magnitude and sign of the charge distribution (σ_0_) and the potential (*ψ*_0_) at the interface between functional layer and the Stern layer. This dissociation or protonation of the groups can be schematically generalized in a manner similar to that of amino acids as


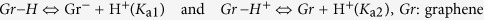


An example for the former case are carboxyl groups which are dissociated at high pH, while the latter case can be thought of as hydroxyl or amine groups which are protonated at low pH. The fractions of dissociated and protonated groups are respectively given by


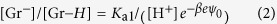



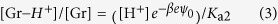


where the square brackets indicate the surface concentration of the respective species, except for [H^+^], which corresponds to the bulk proton concentration^22^. By specifying a maximum charge density (*σ*_max_) at maximum dissociation or protonation, (see Notes/N1 in SI)^26^ we can write the individual charge distributions at the functional layer as


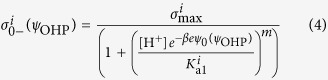



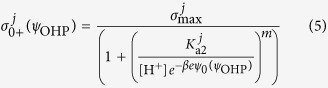


where the superscript index *i* or *j* refers to different kinds of negatively or positively chargeable functional groups respectively each with a corresponding *K*_a_ and *m* specifies the degree of heterogeneity in the distribution of ionizable groups as specified by modified Langmuir-Freundlich theory^27^. By appropriate rearrangement of the terms, the net charge density at the interface can then be compactly written as


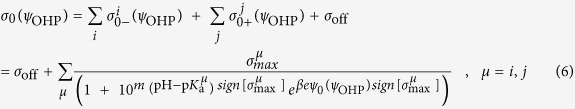


where sign[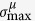] is +1 or −1 for positively or negatively chargeable groups respectively, *σ*_off_ is a charge density offset which is constant over a range of pH and ionic strength^16^. The potential at the interface is given by


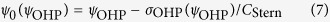


where *C*_Stern_ is the specific capacitance of the Stern layer (thickness *t*_Stern_) given by *ε*_0_*ε*_r−Stern_/*t*_Stern_.

In order to arrive at the charge carrier distribution in graphene, we need to consider that in an experiment an external voltage^22^ is imposed at the graphene-liquid interface. The electrochemical gate voltage (*V*_ecG_) applied through a reference electrode leads to an electrified interface, whose effect on the charge carrier density in graphene is given by


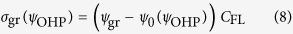



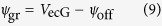


with *ψ*_gr_ referring to the graphene electrostatic potential, *C*_FL_ = *ε*_0_*ε*_r−FL_/*t*_FL_ and *ψ*_off_ is a cumulative offset potential that is independent of pH or ionic strength of the solution. This includes measurement offsets, offset voltage arising due to the choice of the reference electrode and offsets due to the varying filling levels of graphene from impurities or the underlying substrate (the part which is only in contact with graphene and not with the liquid)^28^. With equations (1), (6, 7, 8, 9) we can solve for *ψ*_OHP_ by ensuring charge neutrality using the transcendental equation


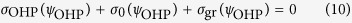


Using the value of *ψ*_OHP_ at every triple {pH, *c*_I_, *V*_ecG_}, the charge-potential distribution at all planes of the graphene-liquid interface can be self-consistently computed. From the experimental perspective, we can extract the gate voltage at the Dirac point (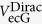) using a field-effect measurement in liquid. The pH and ionic strength *c*_I_ of the liquid can be varied and the Dirac point recorded as a function of pH and *c*_I_. The Dirac point corresponds to the point of minimum charge carrier density^10^,^29^. The same charge neutrality point can also be extracted from the model for a certain pH and *c*_I_ by solving for 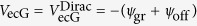, such that *σ*_gr_(*ψ*_OHP_) = 0. The minus sign occurs because the applied gate voltage in an experiment occurs at the reference electrode, whereas in the model it occurs at the graphene plane. Since we restrict here only to the situation at the Dirac point, the gate dependent variation in charge carrier density occurring due to the linear energy dispersion in graphene and the effect of quantum capacitance^30^ are neglected in equations (8) and (9).

Figure 1b presents a map of 
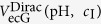
 computed using the above model, while Fig. 1(c) shows the behavior of 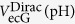 for selected values of ionic strength (IS). For this simulation, we have assumed two sets of ionizable groups of equal charge density maximum – one positive and the other negative – each with a p*K*_a_ of 7 and hence we can talk of an isoelectric point of the surface to be 7. It is apparent that the Dirac point variation at higher pH is compressed at higher ionic strength due to the smaller thickness of the electrical double layer, consistent with previous measurements on graphene^16^. Interestingly, curves in Fig. 1c are found to cross at the pH value corresponding to the isoelectric point of the surface. Figure 1(c) is reminiscent of the pH-dependent charging of a protein around its isoelectric point. The offset voltage *ψ*_off_ is taken as −0.1 V here (Fig. S1 shows another example for *ψ*_off_ = 0), although this may vary from sample to sample due to the spatially varying chemistry of CVD-grown graphene, size of the flake and local charging effects from the underlying substrate. We can exclude this parameter by comparing the pH-Dirac point behavior at 1 M ionic strength to the behavior at the other IS values. Figure 1(d) presents the relative Dirac point shift (blue line) between the curve at 1 M IS and that at 1 mM IS, along with a plot of the net surface charge density (red curves). Here it can be clearly seen that at the pH value corresponding to the isoelectric point, the difference curves exhibit a zero crossing. The sign of this so-called *difference Dirac curve (M-I)* correlates directly with the sign of the net surface charge. We have used the data at 1 M ionic strength as the baseline due to this reason. This is due to the fact that the compressed double layer at high IS leads to an effective interface potential whose absolute magnitude is smaller than the value at low IS. Now, in order to determine the pI experimentally, we measure the Dirac point as a function of pH and IS and deduce difference Dirac curves such as the one in Fig. 1(d). The zero crossing of such a curve directly gives us an estimate of the pI of the surface, while the sign of this curve determines the kind of net surface charge at a given pH.

Before going to the experimental details, it is worth clarifying the definitions of pI/pzc^31^,^32^ used here. Analogous to the terminology in biochemistry (e.g. for proteins) we define the isoelectric point to be the value of pH at which *σ*_0_ = 0, i.e. the net charge of the surface is found to be 0^33^. In the absence of specific adsorption of ions, the pI and pzc values are identical^20^,^23^,^34^. (see Notes/N2 in SI) In the model outlined above, we have not included the effect of specific adsorption of ions^34^ on to the graphene surface. A more rigorous model including this aspect is presented in the supplementary information. For the case of specific adsorption it is expected that the pzc shifts away from the value of the isoelectric point^23^. Upon including specific adorption into our model calculations, the zero crossings of the difference Dirac curves are found to shift as a function of IS, depending on the sign and extent of the adsorbed ions (see discussion in SI – page S1–S4, Figs S1–S3). We exploit this feature to identify the presence or absence of specific ion adsorption in our experiments. Thus, by fitting the measured data to the model, we extract both the pzc and IEP values as well as the nature and extent of ion adsorption at the interface. Nevertheless, the extent of ion adsorption is found to be rather low, yielding a pzc IEP difference of maximally 0.5 pH units for all the cases experimented here.

### The isoelectric point of bare (unmodified/as-prepared) graphene

**Figure 2:**
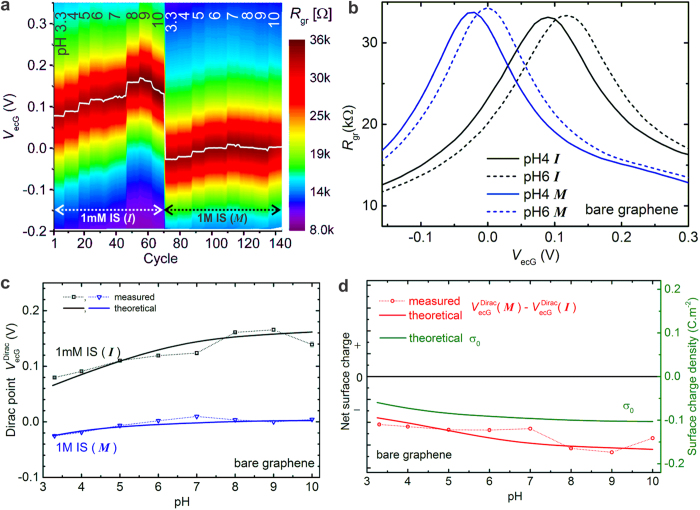
The isoelectric point of bare (unmodified/as-prepared) graphene. (**a**) A 2D-map showing the evolution of gate dependence of graphene resistance (*R*_gr_) as a function of varying pH and ionic strength (*I* − 1 mM, *M* − 1 M ionic strength; e.g. 6*I* refers to a pH 6 solution of 1 mM ionic strength). Every cycle takes around 10 s. The measurement is paused during solution exchange. *V*_ecG_ refers to electrochemical gate voltage applied through a Ag/AgCl reference electrode that is in contact with solution. The white profile superimposed on the 2D-map indicates the position of the Dirac point estimated from the profiles such as in (**b**). (**b**) Line profiles extracted from the 2D-map showing the gate dependence of graphene resistance in four different solutions, where the shift in Dirac point is discernible. (**c**) Measured and calculated Dirac point profiles as a function of pH at 2 different ionic strength values. (**d**) (red curves) measured and calculated difference Dirac curves obtained by subtracting the curve at 1 mM IS from that at 1 M IS (referred to as *M* − *I*). For comparison, the calculated surface charge density as a function of pH (green curve) is also superimposed. The difference curve is used as a measure to infer the sign of net surface charge on graphene. The simulated curves were obtained by assuming a pI of 2.0. See supplementary details for more information on model parameters.

Graphene devices were fabricated by transferring CVD-graphene on to Si/SiO_2_ chips and patterning them using photolithography (See Methods for details)^29^. At the end of the fabrication process, we are left with a contacted graphene flake of size around 2 μm × 2.5 μm (see Fig. S5a), which is exclusively in contact with the solution with all lead electrodes passivated appropriately^35^. In order to ensure that the graphene surface is free of organic and trace metal impurities, we perform a rapid thermal annealing at 600 °C and an electrochemical etching procedure in HCl^12^. The field-effect in liquids is recorded by applying a gate voltage (through a Ag/AgCl reference electrode) and measuring the real part of impedance (*R*_gr_) at a frequency of 1 kHz continuously in buffer solutions of varying pH and ionic strength (see supplementary information for preparation of buffer solutions)^12^. Figure 2(a) shows a typical measurement on bare graphene (as-prepared or unmodified graphene is referred to here as bare graphene) for varying pH values at two different ionic strengths (1 mM: *I* and 1000 mM: *M*). From every cycle of field-effect scan, as shown in Fig. 2(b), we extract the Dirac point (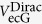 or the gate voltage at resistance maximum), which is also overlaid in Fig. 2(a). The dotted lines in Fig. 2(c) show the dependence of this Dirac point as a function of pH for the two IS values, while that in Fig. 2(d) shows a plot of the *M-I* difference curve representative of the sign of surface charge on graphene.

From Fig. 2(d) it is apparent that the surface is predominantly negatively charged and we do not have a zero-crossing in the measured pH range. Based on this observation it can be concluded that the pI of bare (unmodified or as-prepared) graphene must be less than 3.3. Figure 2(c,d) also include theoretically calculated curves (solid lines) by assuming a pI of 2 for graphene, which are in good agreement with the measured values (see Fig. S4 the measured curves and theoretical fits at all 4 values of IS). This very low pI is consistent with isoelectric point data available on graphite and the frequent observation of negative charges on graphitic surfaces and particles^19^,^36^,^37^. The occurrence of low pI at liquid interfaces with non-polar hydrophobic surfaces (in general) has mainly been attributed to the structure and orientation of water molecules at the double layer^38^,^39^. Moreover, charging of the non-polar material-water interface through specific adsorption of OH¯ ions has also been observed^40^,^41^. The effect of specific adsorption of ions has been included in the theoretical curves in Fig. 2. A good fitting to the measured curves could be obtained by including only anion adsorption with a charge density corresponding to around 7% of the maximum graphene charge and a binding constant of 50 mol^−1^L. This corresponds to an average charge density of around 1 μC/cm^2^ close to the values reported for homogenized alkane emulsions in water (around 5 μC/cm^2^)^41^,^42^. The selective adsorption of anions is attributed to some kind of chemical interaction between the anions (hydroxyl or otherwise) in the solution and graphene^43^. The parameters of specific adsorption are also consistent with the case of functionalized graphene as will be seen later. Another important aspect concerns the effect of the underlying SiO_2_ substrate. The capacitance due to trapped charges in SiO_2_ is much weaker than the double layer capacitance at the graphene-liquid interface^44^. Hence it can be assumed that this effect is rather constant and grasped as an offset voltage in the simulations. On the other hand the silicon oxide surface possesses ionizable groups^45^,^46^, which are indeed present very close to the graphene edges^47^ and whose pH-/IS-dependent (dis)charging may have an effect on the potential landscape of the interface. In the fitted curves in Fig. 2 this contribution is included by taking the maximum charge density due to the silanol groups to be 15% of the maximum charge density on graphene (see supplementary information details).

### Effect of chemical functionalization on the isoelectric point of graphene

**Figure 3:**
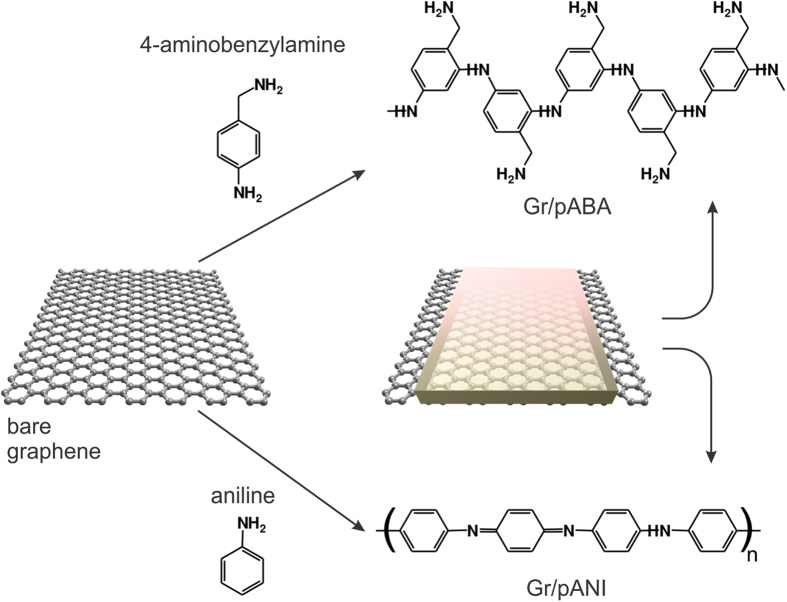
Scheme of electrochemical functionalization of graphene. Functional groups are attached to graphene by electropolymerization of monomeric precursors. Here, two precursors have been evaluated – *4*-aminobenzylamine (ABA) or aniline (ANI), leading to polymeric layers : poly(aminobenzylamine) (Gr/pABA) or polyaniline (Gr/pANI).

Now we turn towards the effect of attaching functional groups on the isoelectric point of graphene surface. For this purpose we choose an electrochemical route since it allows for the versatile attachment of a broad range of chemical moieties by simple variation of experimental parameters^48^,^49^,^50^. Moreover, the same field-effect setup in liquids can be used for performing the electrochemical modification (ECM)^51^. Here, we have chosen to compare the effect of two different precursors *4*-aminobenzylamine (ABA) and aniline (ANI)^52^ Both of them can be electropolymerized over graphene in aqueous solutions and allow for a control of the thickness of the electropolymerized layers through the parameters such as electropolymerization voltage, concentration and time. Figure 3 shows a scheme of the electropolymerization strategy. AFM images (see Fig. S5) before and after attachment of the polymers are used to estimate the thickness of the attached moieties. Raman spectra (see Fig. S5) are used to infer that the polymer does not introduce covalent bonds to graphene ensuring that the favorable electronic properties of underlying graphene remain unaffected^52^. Although the chemical compositions of these two polymers have some similarities, the p*K*_a_ of ABA is 8.5, in contrast to that of ANI, which is 4.2^53^,^54^.

**Figure 4:**
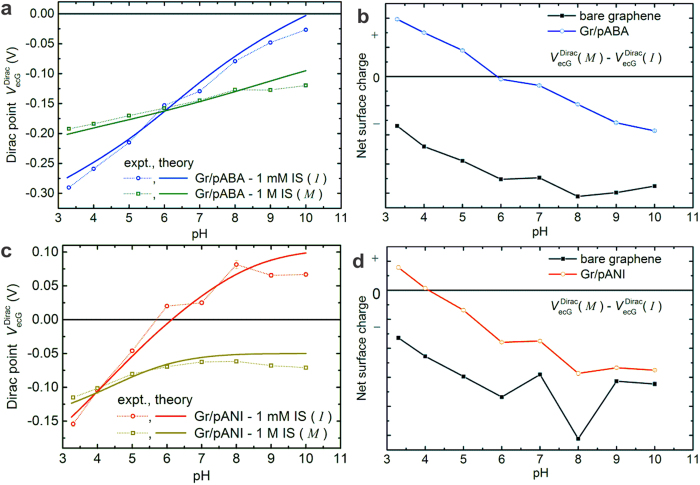
The isoelectric point of functionalized graphene for 3 nm-thick layers of pABA (a,b) and pANI (c,d). (**a**) Measured and calculated Dirac point profiles for Gr/pABA as a function of pH at 1 mM and 1 M ionic strength. (**b**) Difference Dirac curves (obtained by subtracting the two curves in (**a**) before (black) and after modification (blue) giving an estimate of IEP as around 6 for Gr/pABA. (**c**) Measured and calculated Dirac point profiles for Gr/pANI as a function of pH at 1 mM and 1 M ionic strength. (**d**) Difference Dirac curves before (black) and after modification (red) giving an estimate of IEP to be around 4. The pI of bare graphene is less than 3.3 in both cases. See supplementary information about the details of model parameters (Figs S6–S8).

Figure 4(a) presents the pH-dependent Dirac point behavior of a graphene device at two different ionic strength values (1 mM and 1 M) after attachment of 3 nm-thick functional layers of poly(aminobenzylamine) (pABA). Figure 4(b) shows the *M-I* difference Dirac curve (blue) obtained from this measurement along with the difference Dirac curve before modification (black curve). The curve before modification does not have a zero crossing consistent with the discussion that the pI/pzc of graphene is less than 3.3. However, after modification the difference curve exhibits a clear zero crossing at around a pH of 6, which is attributed to be the approximate pI of the functionalized surface. In order to obtain support for this claim, we have used the model to simulate the presence of pABA by introducing an additional type of ionizable group with a charge density that is around 1.2 times that of graphene (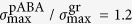 for this device) and a p*K*_a_ of 8.5. The effect of the silanol groups at (15%) and that of specific ion adsorption (7%) remain the same as for bare graphene. The fitted curves are presented as solid lines in Fig. 4(a) where it can be seen that a qualitatively good agreement is obtained. From this model we extract a pI of 5.98 and pzcs of 5.98 and 5.6 at an IS of 1 mM and 1 M respectively, while the experimental zero crossing of *M-I* difference curve occurs at 5.92. Further support for this estimation is obtained from Fig. S6 presenting the complete dataset, along with the simulated curves for all the 4 IS values. Almost all the aspects, including the shift in the zero crossing (which can be unambiguously attributed to specific adsorption of anions) are well-reproduced using the model. In order to confirm that we are indeed modifying the pI of the surface we have repeated the same measurement for the case of polyaniline (pANI). The theoretical curves are modelled by setting the p*K*_a_ of the type of ionizable group to 4.2 (instead of 8.5 as done before). The results collected in Fig. 4(c,d) (see also Fig. S7), show a clear agreement between theory and experiment. The model gives a pI value of 3.85 and pzcs of 3.82 and 3.38 at 1 mM and 1 M IS respectively, while the experimental *M-I* zero crossing occurs at 4.05. Further details of the model parameters are discussed in supplementary information. Based on these observations, we will now use the pH value of zero crossing of the *M-I* difference Dirac curve directly as an approximate estimate of the surface pI.

**Figure 5:**
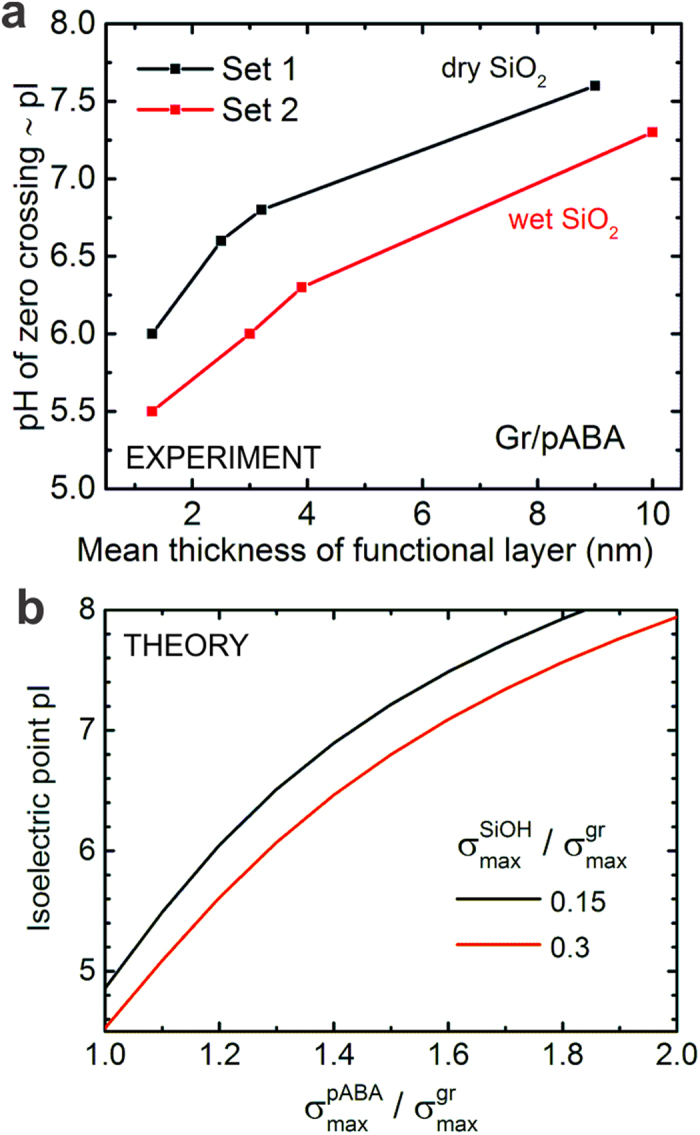
Effect of the density of functionalities and substrate on the isoelectric point of graphene. (**a**) Evolution of the isoelectric point (IEP) of functionalized graphene as a function of the average thickness (obtained from AFM data) of the attached functionalities (pABA) measured on two different kinds of Si/SiO_2_ substrates. Set 1 refers to dry oxide obtained by thermal oxidation while Set 2 refers to wet oxide obtained by wet chemical etching. Substrates of set 2 exhibit a higher density of silanol groups in comparison to set 1. (**b**) Simulated curve showing the evolution of IEP with increasing density of attached functionalities (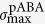) for two different kinds of substrates each with a different density of silanol groups (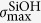). The densities are input to the model relative to the intrinsic surface density of graphene (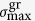). It is apparent that the ionizable groups on the underlying substrate bring mainly a constant offset to the surface charge on functionalized graphene.

The most important conclusion from the foregoing discussion is that the functionalized graphene surface assumes an effective pI that is a weighted balance between the p*K*_a_ (or pI) of bare (unmodified) graphene, the attached moieties and to some extent the underlying substrate. The weighting factor is then determined by the density of ionizable sites (see Fig. S8 for a comparison of pABA and pANI, where the effect of a change in p*K*_a_ is presented). It is worth mentioning that we are modifying the acid-base equilibrium of the graphene-liquid interface through electrochemical functionalization and hence the pI is modified in this process. The change in pzc is a consequence of this modification and sets in only when we have specific adsorption of ions. The immediate question that comes up is to see the effect of increasing thickness of the functional layers on the isoelectric point of functionalized graphene. For this purpose, we have repeated the above experiment for varying thickness of the polymer layer (see Methods) in many different devices. We first measure the pH-IS-behaviour of unmodified graphene at 4 different IS values, perform the electrochemical functionalization and repeat the pH-IS-measurement followed by an estimation of the layer thickness using AFM. Figure 5(a) plots a summary of these measurements (black curve, Set 1) for varying polymer thickness. It is apparent that we see a clear trend of increasing pI (and pzc) as the thickness of the functional layer increases on the graphene surface (see Fig. S9 for data at other IS values). This is consistent with an increase in the density of attached functional groups as exemplified by the simulated black curve in Fig. 5(b).

### Effect of substrate

Another important aspect concerns the role of SiO_2_ on the value of pI of the functional interface. For this purpose, we have performed a similar set of measurements on another kind of substrate (referred to as Set 2), namely an oxide prepared using a wet etching procedure (the previous devices were measured on dry oxide). The wet oxide results in a comparatively higher density of silanol groups^55^,^56^. The values of pI measured on this substrate for varying thickness of the functional layer are compared in Fig. 5(a) (red curve, Set 2). It is apparent that we have a downward shift in the isoelectric point by around 0.4 pH units for the same thickness of functional layer. This can be explained by considering that the higher density of negatively charged ionizable groups on the wet oxide leads to a larger weighting of the contribution from silanol groups thereby offsetting the pI of the surface to lower pH values. This can be modeled in a straightforward manner by just varying the relative contribution of the maximum charge density due to silanol groups with respect to that of graphene. (see Notes/N3 in SI) The resulting pI variation presented in Fig. 5(b) (red curve) agrees quite well with the trend of measured data in Fig. 5(a). From the ongoing discussion we can conclude that the underlying substrate brings in mainly a constant offset to the net pI/pzc achievable through chemical functionalization without directly affecting the modulation of the pI that can be attained by a continuous variation in the thickness of the functional layer. This offset can be set externally either by using a substrate of different charge density or by using alternative substrates that exhibit a different pI (such as hBN or polymer)^57^.

### Tuning the isoelectric point of graphene

**Figure 6:**
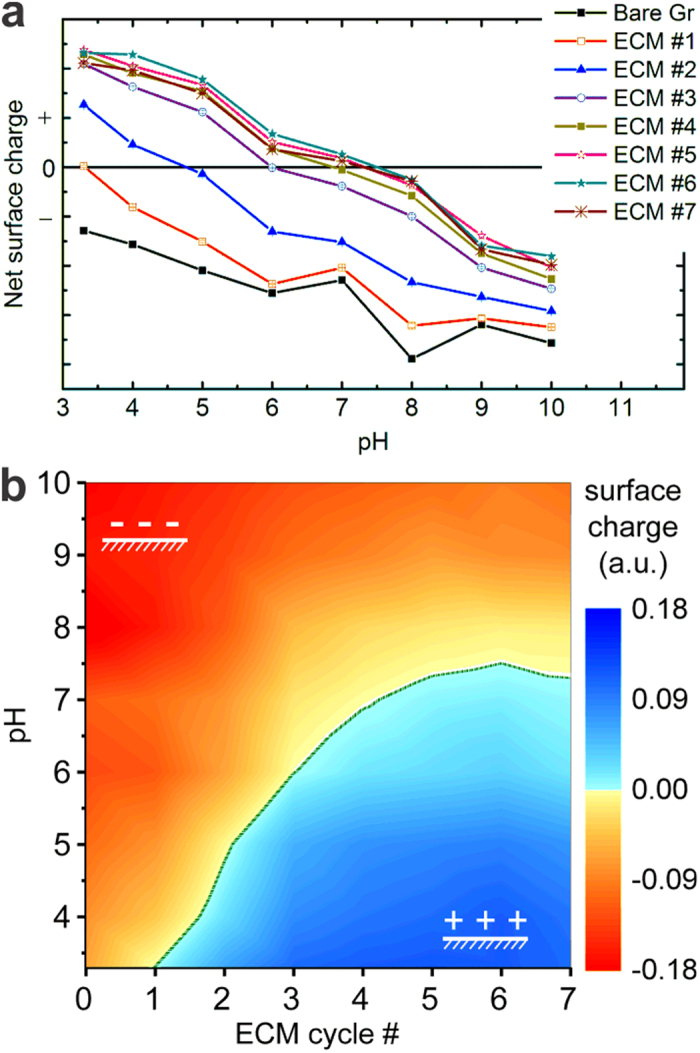
Tuning the isoelectric point of graphene by consecutive electrochemical modification (ECM) using 4-aminobenzylamine. The difference Dirac curves before and after every ECM are plotted as a function of pH in (**a**), while the same curves are shown in (**b**) as a 2D-map in order to exemplify the evolution of the isoelectric point. The IEP occurs along the green line between the blue and orange regions and could be varied from less than 3 up to slightly more than 7 in this case. With every ECM, the density of attached groups increases and leads to a modulation of the surface charge. (**b**) gives a clear picture of the surface charge being negative in the orange region, and positive in the blue region allowing us to identify the set of parameters needed to obtain a desired pI within the tunable range. (see Fig. S10 for raw Dirac point profiles).

Finally, we demonstrate that we can continuously tune the value of pI of the functionalized graphene surface by exploiting the versatility of the electrochemical modification protocol. For this purpose, we perform the characterization of pH-IS-behaviour at the initial stage and after every consecutive electrochemical modification. With every modification the density of functional groups on the graphene surface increases resulting in a shift of the isoelectric point towards higher pH values. We perform this series of experiments continuously without drying the sample in order to ensure that the functional groups remain intact during the entire measurement series. Figure 6(a) presents the difference Dirac curves at every step, while Fig. 6(b) shows the evolution of the surface charge with every cycle of electrochemical modification as a 2D-map (complete measured raw dataset in Fig. S10). These results exemplify the capability to continuously vary the pI from less than 3 up to around 7.5 for this device. In this manner, we have designed a sophisticated methodology using which we can set the pI of the graphene surface to a desired value for a given type of chemical functionality (here amino groups) allowing for the possibility to astutely engineer the charge distribution of the graphene surface for an application of interest. Interestingly, the simulation (Fig. S8) indicates that we should be able to attain a pI that is higher than that of what we see in Fig. 6. We attribute this saturation to the inability to get a complete intact coverage of the functional groups on graphene most likely due to steric hindrances and the difficulty to obtain homogeneous charge transfer to the monomer over the entire area^58^. Moreover, the polymer may be porous allowing direct access of the solution to the graphene surface. Some support for this fact is obtained from AFM images (see Fig. S5), where one observes that the attached moieties form a rugged and coarse pattern rather than a perfectly smooth layer.

## Concluding Remarks

In conclusion, we have presented a strategy to estimate the isoelectric point of bare (unmodified/as-prepared) and functionalized graphene. The surface charge behavior is determined by the weighted average of acid-base properties of the ionizable groups on unmodified or functionalized graphene. While as-prepared CVD-graphene exhibits a pI below 3.3, we could tune this value to higher levels by a judicious attachment of a controlled density of ionizable groups on the graphene surface. In this manner the graphene surface can be rendered positively or negatively charged with the same type of chemical functionality at a given pH. Using a theoretical model we could also show that specific adsorption of ions indeed occurs at the graphene-liquid interface albeit with a minor effect on the capability to tune the net surface charge density of functionalized graphene. The presented theory and the methodology can be used to identify changes in pI/pzc using other chemical functionalization strategies and thereby serves as a versatile platform for engineering the interfacial properties of graphene for a targeted application.

## Methods

### Graphene devices

First, pre-patterned electrode lines with 4 micron gaps are prepared on 4” Si/SiO_2_ wafers using photolithography. Two kinds of wafers have been used – one with a dry oxide of 575 nm thickness obtained by thermal oxidation (Set 1) and the other obtained by wet chemical etching with an oxide thickness of 500 nm. CVD-graphene obtained either commercially (Graphene Supermarket Inc.) or by a peel-off process Ref. 29 was cut into rectangular pieces (typically 1 cm × 0.5 cm) and a solution of poly(styrene) (PS) (50 mg/mL in toluene) was spotted over the copper foil (PS/graphene/copper/graphene) and dried at 75 °C for 10 minutes. After the deposition of PS, the underlying copper was removed by etching in a solution of hydrochloric acid with added hydrogen peroxide (1.4 M HCl + 0.5 M H_2_O_2_) leading to removal of copper in less than 10 minutes. Then, graphene was transferred to Si/SiO_2_ chips with the prepatterned Ti/Pt electrode lines and baked in the oven at 95 °C for 20 minutes before removal of PS using toluene. The patterning of the flakes (of size 2 μm × 2.5 μm) was also performed by photolithography. For this purpose, 10 nm of copper was first evaporated on graphene to be used as a sacrificial protective layer in order that the photoresist does not come in direct contact with the graphene surface. Following this, the structures are patterned using a positive process using the resist S1805 (Microposit). After exposure and development the unprotected regions are removed using mild oxygen plasma etching. After this, the resist and the remaining copper are removed in *N*-ethylpyrrolidone and HCl/H_2_O_2_ respectively. Subsequently we do another round of lithography to deposit SiO_2_ by thermal evaporation in order to passivate all electrode lines using an insulating layer. This ensures that graphene is exclusively in contact with the solution. Following this an electrochemical etching procedure was carried out in HCl in order to remove trace metal impurities^12^ present on patterned graphene. The chips were then annealed at 585 °C for 60 seconds in argon atmosphere to remove organic impurities and to improve the contact between graphene and the electrodes/substrate.

### Electrical measurements

The field-effect measurements in liquid were carried out in a PDMS (poly(dimethylsiloxane)) channel placed on the graphene device to hold the liquid (volume is approx. 200 μL). The gate voltage was applied using a Ag/AgCl reference electrode (WPI Inc.). 0 mV vs Ag/AgCl corresponds to 50 ± 5 mV versus SCE measured in 0.1 M KCl. The impedance of the device was measured using an LCR Meter (Agilent) at 1 kHz. The gate voltage was continuously swept in a fixed range and the real part of the impedance recorded to obtain the data as in Fig. 1. In some experiments the Dirac point showed a sudden drift, which is identified and corrected by repeating the recording of the response at pH3.3 at the end of every set of measurements at a given IS. The buffers with varying pH and ionic strength were carefully prepared as discussed in SI and stored in the fridge as 50 mL stock solutions.

### Electrochemical modification

The electrochemical modification (ECM) to deposit the functional layers was carried out via oxidative polymerization. The same PDMS channel was filled up with the precursor solution and an Ag/AgCl electrode was used as the reference electrode. The same electrical setup mentioned above was used to perform the modification^59^. The solutions with the precursors were prepared just before the modification. For Gr/pABA, an aqueous solution of 10 mM ABA and 100 mM LiClO_4_ was introduced into the microwell and the voltage at graphene was swept from −0.2 to +0.7 V against the Ag/AgCl reference for 1 cycle. For Gr/pANI, a mixture of 10 mM ANI and 100 mM LiClO_4_ was used, and the voltage was swept from −0.2 to +0.8 V for 4 cycles. For tuning the pI, a series of electrochemical polymerization runs were carried out, using ABA as precursor. For the first 3 modifications, 0.1 mM, 1 mM and 10 mM ABA were used at a voltage of 0.7 V for 1 cycle. The subsequent modifications were also carried out at 0.7 V and 10 mM ABA but increasing the number of cycles from 3 up to 12.

### Raman and AFM Images

Raman spectra of graphene samples on Si/SiO_2_ chips were measured using an NT-MDT NTEGRA system or a S&I MonoVista system, with a laser excitation of 632.8 nm at a power of 2.7 mW or 1 mW respectively. This system was equipped with a 520 mm monochromator and a 600 l/mm grating. The acquisition time was 5s. AFM images were obtained using a Digital Instruments Veeco Dimension III in tapping mode. The acquired AFM images were processed using Gwyddion software.

### Modeling

All equations are modelled symbolically and solved numerically in Mathematica 10. The fitting of model parameters to the data was performed in a semi-automatic manner by programmatically varying the parameters in a given range and minimizing the deviation between the measured and simulated curves.

## Additional Information

**How to cite this article**: Zuccaro, L. *et al.* Tuning the isoelectric point of graphene by electrochemical functionalization. *Sci. Rep.* **5**, 11794; doi: 10.1038/srep11794 (2015).

## Supplementary Material

Supplementary Information
