# Supplementary material for: Tuning the isoelectric point of graphene by electrochemical functionalization
Source: Sci Rep. 2015 Jul 2;5:11794. doi: 10.1038/srep11794 (PMC4488746; doi:10.1038/srep11794)
Supplement: Supplementary Information [file srep11794-s1.pdf]

Tuning the isoelectric point of graphene by electrochemical functionalization

Laura Zuccaro[1], Janina Krieg[1§], Alessandro Desideri[2], Klaus Kern[1,3] and Kannan Balasubramanian[1]*

[1] Max Planck Institute for Solid State Research, D-70569 Stuttgart, Germany.
[2] Dept. of Biology, University of Rome Tor Vergata, I-00133 Rome, Italy.
[3] Institut de Physique de la Matière Condensée, Ecole Polytechnique Fédérale de Lausanne, CH-1015 Lausanne, Switzerland.
[§] *Current Address : GSI Helmholtz Center for Heavy Ion Research, 64291 Darmstadt, Germany.*

* *Corresponding author e-mail* : b.kannan@fkf.mpg.de

SUPPLEMENTARY INFORMATION

The graphene liquid interface with specific ion adsorption

In order to include specific adsorption of ions, we introduce the inner Helmholtz plane (IHP) within the Stern layer, where the ions get immobilized as shown in fig. S2(a). The IHP is taken to be at a distance (t_{IHP}) from the functional layer. The specific capacitance of this inner layer is given by $C_{\mathrm{IL}} = \varepsilon_0 \varepsilon_{\text{r-IL}} / t_{\mathrm{IHP}}$, where $\varepsilon_{\text{r-IL}}$ is the dielectric constant of the region between the functional layer and IHP. We can now differentiate two cases of specific adsorption namely due to electrostatic interaction with the charges in the functional layer (case 1) or due to a direct chemical interaction with (functionalized) graphene (case 2).

Case 1: Specific ion adsorption due to the functional layer

For this case, the equilibrium for ion adsorption of type $\mathcal{C}^+\mathcal{N}^-$ ($\mathcal{C}^+$ referring to cations, $\mathcal{N}^-$ referring to anions) can be written as

$Gr\text{-}FL^- + \mathcal{C}^+ \Leftrightarrow Gr\text{-}FL^-\mathcal{C}^+ \quad (K_\mathcal{C})$ and

$Gr\text{-}FL^+ + \mathcal{N}^- \Leftrightarrow Gr\text{-}FL^+\mathcal{N}^- \quad (K_\mathcal{N})$, *Gr* : graphene, *FL* : functional layer

with $K_\mathcal{C}$ and $K_\mathcal{N}$ refer to binding constants for the association/dissociation of univalent ions with the functional layer. By assuming that the concentrations of $\mathcal{C}^+$ and $\mathcal{N}^-$ are directly related to the total ionic strength c_{I}, the charge density occurring at the IHP can be written in a form similar to equations (4-6) as

$$\sigma_{\mathrm{IHP}}^{\mathrm{es}}(\psi_{\mathrm{OHP}}) = \frac{\left|\sum_i \sigma_{0-}^i(\psi_{\mathrm{OHP}})\right|}{\left(1+K_\mathcal{C}\, c_{\mathrm{I}}\, e^{-\beta e \psi_{\mathrm{IHP}}(\psi_{\mathrm{OHP}})}\right)} - \frac{\left|\sum_j \sigma_{0+}^j(\psi_{\mathrm{OHP}})\right|}{\left(1+K_\mathcal{N}\, c_{\mathrm{I}}\, e^{+\beta e \psi_{\mathrm{IHP}}(\psi_{\mathrm{OHP}})}\right)} \qquad (SIeq1)$$

with the first term referring to cations and the second to anions. Here we have assumed that there is only one binding constant each for the cations and anions although the individual type of ionizable group (represented by *i* and *j*) may be different. This detail may however be easily incorporated by introducing functional group-specific binding constants $K_{\mathcal{C}}^{i}$ and $K_{\mathcal{N}}^{j}$ and performing the summation over the entire fractional term instead of doing it only for the numerator. The potential at IHP is given by

$$\psi_{\text{IHP}}(\psi_{\text{OHP}}) = \psi_{\text{OHP}} - \sigma_{\text{OHP}}(\psi_{\text{OHP}})/C_{\text{Stern}} \qquad (SIeq2)$$

with $\sigma_{\text{OHP}}(\psi_{\text{OHP}})$ given by equation (1). In equation (SIeq1) the σ_0 terms are dependent on ψ_0, which in turn depends on $\sigma_{\text{IHP}}^{\text{es}}$. So, we have to introduce ψ_0 as an unknown in addition to ψ_{OHP}. Now the charge density at the functional layer σ_0 is given by equation (6) by replacing $\psi_0(\psi_{\text{OHP}})$ with just ψ_0. Finally the charge and potential in graphene are related by equations (8) and (9), again by replacing $\psi_0(\psi_{\text{OHP}})$ with ψ_0. Now, we have two unknowns ψ_{OHP} and ψ_0 and they can be obtained at every triple $\{\text{pH}, c_{\text{I}}, V_{\text{ecG}}\}$ by solving the system of equations

$$\sigma_{\text{OHP}}(\psi_{\text{OHP}}) + \sigma_{\text{IHP}}^{\text{es}}(\psi_{\text{OHP}}, \psi_0) + \sigma_0(\psi_{\text{OHP}}, \psi_0) + \sigma_{\text{gr}}(\psi_0) = 0 \qquad (SIeq3)$$

$$\psi_0 - \psi_{\text{IHP}}(\psi_{\text{OHP}}) + \frac{\sigma_{\text{IHP}}^{\text{es}}(\psi_{\text{OHP}}, \psi_0)}{C_{\text{IL}}} = 0 \qquad (SIeq4)$$

<u>*Case 2: Specific ion adsorption due to chemical interaction with graphene*</u>

In this case, we can write the equilibrium for the binding of ions as

$Gr + \mathcal{C}^+ \Leftrightarrow Gr\text{-}\,\mathcal{C}^+ \quad (K_{\mathcal{C}}^{\text{gr}})$ and

$Gr + \mathcal{N}^- \Leftrightarrow Gr\text{-}\,\mathcal{N}^- \quad (K_{\mathcal{N}}^{\text{gr}})$,

with $K_{\mathcal{C}}^{\text{gr}}$ and $K_{\mathcal{N}}^{\text{gr}}$ referring to binding constants for the direct chemical interaction of the ions with (functionalized) graphene. This interaction leads to accumulation of charges again at the IHP similar to case 1. However, the position of the IHP in this case may be different from the previous one. This can however be modelled directly by differing specific capacitances of the inner layer (C_{IL}). The charge density in this case is given by

$\sigma_{\text{IHP}}^{ii}(\psi_{\text{OHP}}) = \sigma_{\max}^{ii}(\Theta_+ - \Theta_-)$ with

$$\Theta_+^{-1}(\psi_{\text{OHP}}) = 1 + \frac{1}{K_{\mathcal{C}}^{\text{gr}} c_{\text{I}}\, e^{-\beta e \psi_{\text{IHP}}(\psi_{\text{OHP}})}} \text{ and } \Theta_-^{-1}(\psi_{\text{OHP}}) = 1 + \frac{1}{K_{\mathcal{N}}^{\text{gr}} c_{\text{I}}\, e^{+\beta e \psi_{\text{IHP}}(\psi_{\text{OHP}})}} \qquad (SIeq5)$$

with ψ_{IHP} given by equation (SIeq2) and σ_{OHP} and ψ_{OHP} related by equation (1). $\sigma_{\max}^{ii}$ is a charge density denoting the maximum density of ions that may adsorb at the interface and Θ_+ and Θ_- represent the coverage through cations and anions respectively. The charge density (σ_0) at the functional layer is given by equations (4-6) with the potential given by

$\psi_0(\psi_{\mathrm{OHP}}) = \psi_{\mathrm{IHP}}(\psi_{\mathrm{OHP}}) - \sigma^{ii}_{\mathrm{IHP}}(\psi_{\mathrm{OHP}})/C_{\mathrm{IL}}$ (*SIeq6*)

Equations (8) and (9) are still valid at the graphene plane and we can obtain ψ_{OHP} by solving the transcendental equation

$\sigma_{\mathrm{OHP}}(\psi_{\mathrm{OHP}}) + \sigma^{ii}_{\mathrm{IHP}}(\psi_{\mathrm{OHP}}) + \sigma_0(\psi_{\mathrm{OHP}}) + \sigma_{\mathrm{gr}}(\psi_{\mathrm{OHP}}) = 0$ (*SIeq7*)

In both the cases we have assumed that the density of adsorbed ions ($\sigma^{\mathrm{es}}_{\mathrm{IHP}}$ or $\sigma^{ii}_{\mathrm{IHP}}$) occurs separately at the IHP. Alternatively, they can be considered to occur directly at the plane of the functional layer and the net charge on the functional layer taken as a sum of the charge due to ionizable groups and due to specifically adsorbed ions. We have simulated both these cases and we did not see a difference in the estimated values of pI / pzc, most likely because the specific capacitances C_{Stern}ad C_{IL} (see below) are very similar. We have discarded multivalent ions here to keep the treatment simple. The goal here is to get a qualitative agreement of the measurements with the model, which is already achieved using this treatment, as will be seen later.

Figue S2(b) presents the results of the Dirac point modulation (as a function of pH and ionic strength) by including specific adsorption of ions. For this simulation all the parameters are same as that of figure 1 except that specific ion adsorption is included. For this case we have taken that only anions interact with the graphene surface (i.e. $K^{\mathrm{gr}}_{\mathcal{N}} = 50$ mol^{-1}L and $K^{\mathrm{gr}}_{\mathcal{C}} = K_{\mathcal{C}} = K_{\mathcal{N}} = 0$). In fig.S2(c) it is apparent that the pH-dependent Dirac point curves do not cross at the same pH value. Fig. S2(d) presents the difference curves for the three different ionic strengths (1 mM, 10 mM and 100 mM) with respect to the reference curve at 1M IS. It is apparent here that the zero crossings shift to lower pH as the ionic strength reduces. The net surface charge density is overlayed over these curves, where it can be seen that the difference curve (1 M – 1 mM) gives a good estimate of the position of pI of the surface.

In figure S3, we compare the case of cations versus anions and show that in the case of cations adsorbing on the surface, the shift in the zero crossing is towards higher pH. An important consequence is that by analyzing the pH-dependent difference Dirac curves for at least 3 different ionic strength values we can unambiguously estimate the nature (cations / anions) and extent of specific ion adsorption in addition to the position of IEP and pzc. The *M-I* difference Dirac curve gives a rough idea of the position of pI. In order to arrive at exact pI and pzc values for a specific experimental data set, we first fit the data to the model

including specific adsorption. The pzc values are obtained by analyzing the charge density profiles σ_0 and σ_{IHP} as a function of pH at every value of IS. The pI value is obtained by finding the zero crossing of the net surface charge density σ_0 in the absence of specific ion adsorption.

Model Parameters

Here, we present a discussion of the model parameter values, which yield the simulated curves in the presented figures. The most important parameter is the pK_a of the individual ionizable groups which are as follows: p$K_\mathrm{a}^{\mathrm{gr}}$ = 2, p$K_{\mathrm{a1}}^{\mathrm{SiOH}}$ = -2, p$K_{\mathrm{a2}}^{\mathrm{SiOH}}$ = 6 [1,2] and for the attached functional groups p$K_\mathrm{a}^{\mathrm{ABA}}$ = 8.5 and p$K_\mathrm{a}^{\mathrm{ANI}}$ = 4.2. The next important parameter is the corresponding maximum charge density ($\sigma_{\mathrm{max}}^{i}$ or $\sigma_{\mathrm{max}}^{j}$ in equations 4-6), which signifies the relative contribution ("weighting factor") of the various functionalities to yield the net surface charge. We set this density to be 1 for graphene and vary the densities of other functionalities relative to this value. SiO_2 has two sets of ionizable groups, where the protonation with p$K_{\mathrm{a2}}^{\mathrm{SiOH}}$ has a slightly lower density. We arrive at 0.15 for p$K_{\mathrm{a1}}^{\mathrm{SiOH}}$ (15% as discussed in main text) and 0.135 for p$K_{\mathrm{a2}}^{\mathrm{SiOH}}$ after iterative fitting of all the measured data under the condition that this factor is the same on all samples. Using these values, the pI of the SiO_2 substrate alone is 3.4 consistent with values reported in the literature. The *K* values for specific ion adsorption are discussed above and were the same for all the simulations. The relative charge densities ($\sigma_{\mathrm{max}}^{\mathrm{ABA}}$ and $\sigma_{\mathrm{max}}^{\mathrm{ANI}}$) are allowed to vary in order to fit the modelled curves with the measured data. This value is found to be 1.2 for pABA data in fig. 4(a) and 1.5 for pANI in fig. 4(b). The charge density for specific adsorption $\sigma_{\mathrm{max}}^{ii}$ was found to be 0.07 (7% as discussed in main text, but varies slightly from 6 to 8% from one device to the other). Taking all this together we can specify the following parameter set for Gr/pABA: $\mathrm{p}K_\mathrm{a} = [2, -2, 6, 8.5]$ and $\sigma_{\mathrm{max}} = q_0[-1, -0.15, +0.135, +1.2]$, with the individual elements for graphene, SiOH, $SiOH_2$ and pABA respectively and $q_0 = 0.88\, e/(\mathrm{nm})^2$. 0.88 is a factor that is specific to the sample and accounts for sample-to-sample variations in the total number of ionizable sites. [3] The offset charge density σ_{off} is 0 in all the simulations here, while the offset voltage (ψ_{off}) varies according to the sample – it is -35 mV in fig 2 / S4, -150 mV in fig.4a / S6 and -115 mV for fig. 4b / S7. The hetereogeneity factor (*m* from Langmuir-Freundlich isotherm) is found to be 0.5 for bare graphene, 0.3 for Gr/pABA and 0.8 for Gr/pANI. The difference arises most likely due to the chemical nature of the polymer with pANI exhibiting a rather homogeneous chemical structure in comparison to pABA. This

parameter mainly affects the shape of the pH-dependent Dirac point curves and does not affect the position of the zero crossing of the difference curves. Finally, we come to the capacitances of the different layers. They were found to be $C_{\mathrm{FL}} = 2\ \mu\mathrm{F/cm}^2$, $C_{\mathrm{IL}} = 200\ \mu\mathrm{F/cm}^2$ and $C_{\mathrm{Stern}} = 240\ \mu\mathrm{F/cm}^2$for all the simulations except for fig. 6 and fig. S9(b). For these two cases the capacitances vary slightly due to a change in the thickness and / or dielectric constant of the Stern and functional layers as the polymer layer grows. The capacitances for the inner and the Stern layers are consistent with previously reported values for semiconductor oxide-liquid interfaces. [2,4] The capacitance of the functional layer is consistent with a dielectric constant ($\varepsilon_{\text{r-FL}}$) of around 7 for a thickness (t_{FL}) of 3 nm. For a variation in C_{FL} in the range of 0.2 to 20 μF/cm^2 and for a variation in C_{IL} and C_{Stern} in the range of 100 – 500 μF/cm^2 only the shape of the pH-dependence of Dirac point is found to change without affecting the pH value of zero crossing of the difference curves. The common parameters of the simulation in fig.1, S1, S2 and S3 are : pK_a = [7, 7], $\sigma_{\max} = q_0[-1,1]$, $q_0 = 0.5\ e/(\mathrm{nm})^2$, m = 0.5 and similar capacitances as given above. The other parameters are above.

Buffer solutions

In order to carry out the planned experiments it is necessary to carefully prepare a range of buffers over a broad pH range. However, precaution has to be taken to ensure that the buffers do not directly interact with graphene or the surface functional groups. Moreover, the buffer strength and most importantly the buffer capacity has to be kept as constant as possible in order to avoid any inhomogeneous charging of the ionizable groups [5] or to avoid unnecessary shifts in the transfer curves of the graphene devices. In order to prepare buffers including all these considerations we have accurately calculated the composition of the buffers. Before we decide for a specific buffer composition, we ensure that the direct interaction with graphene is minimal by running the entire pH-IS cycle as in figure 2. Specific buffer compositions which interact with graphene lead to unrealistic shifts in the position of Dirac point or bring in irreversible changes to the transfer characteristics. These buffers are iteratively replaced with alternate buffer composition to arrive at the final set of buffer compositions. The buffer concentration is in the range of 1-3 mM for all ionic strengths. The total ionic strength is adjusted using NaCl. The buffer capacity is in the range of 1-5 mM for all the buffers used in this study. The various buffers used were: phosphate (pH 3.3, 4, 6, 7), acetate (pH 5), Tris (pH 8) and carbonate (pH 9.2 and 10). The pH values were verified using a pH meter and the

variation is found to be less than ±0.1 pH units for the lower ionic strengths and ±0.3 pH units for 1M ionic strength.

NOTES

[N1] Typically such ionizable groups on SiO_2 in silicon ISFETs have been modelled based on the site-binding theory assuming that the total amount of ionizable sites remains constant. [5,6] Moreover, the silanol groups are treated as amphoteric groups. In contrast, the attached functionalities on graphene are not amphoteric and hence we model this by differing densities of the attached functionalities, and are specified by their relative strength with respect to that of graphene. [3,7] Using this formulation we obtain a rather good agreement in all of our measured devices.

[N2] It is worth mentioning that an opposite definition of IEP / pzc is very often used in the area of colloids, that rely on zeta potential measurements. [8,9] The presented situation is to be clearly distinguished with experiments on zeta potential measurements where the IEP is often said to be at a pH at which the net charge at the shear plane is zero. In our experiments we clearly do not have a shear plane because of the complete absence of flow and an interface such as that is absent here. Irrespective of the definition of pzc / IEP we are able to identify both situations using our presented model and measured results.

[N3] Note that we can however not correlate directly the density of functional groups to the thickness of the functional layer

Figure S1. Simulated charge-potential relationships at the graphene-liquid interface. Same as in figure 1 except that $\psi_{\mathrm{off}} = 0$. In (d) all the difference curves are plotted where it can be seen that the zero crossing pH for all of them occur at the pH value corresponding to the isoelectric point taken for this case in the simulations.

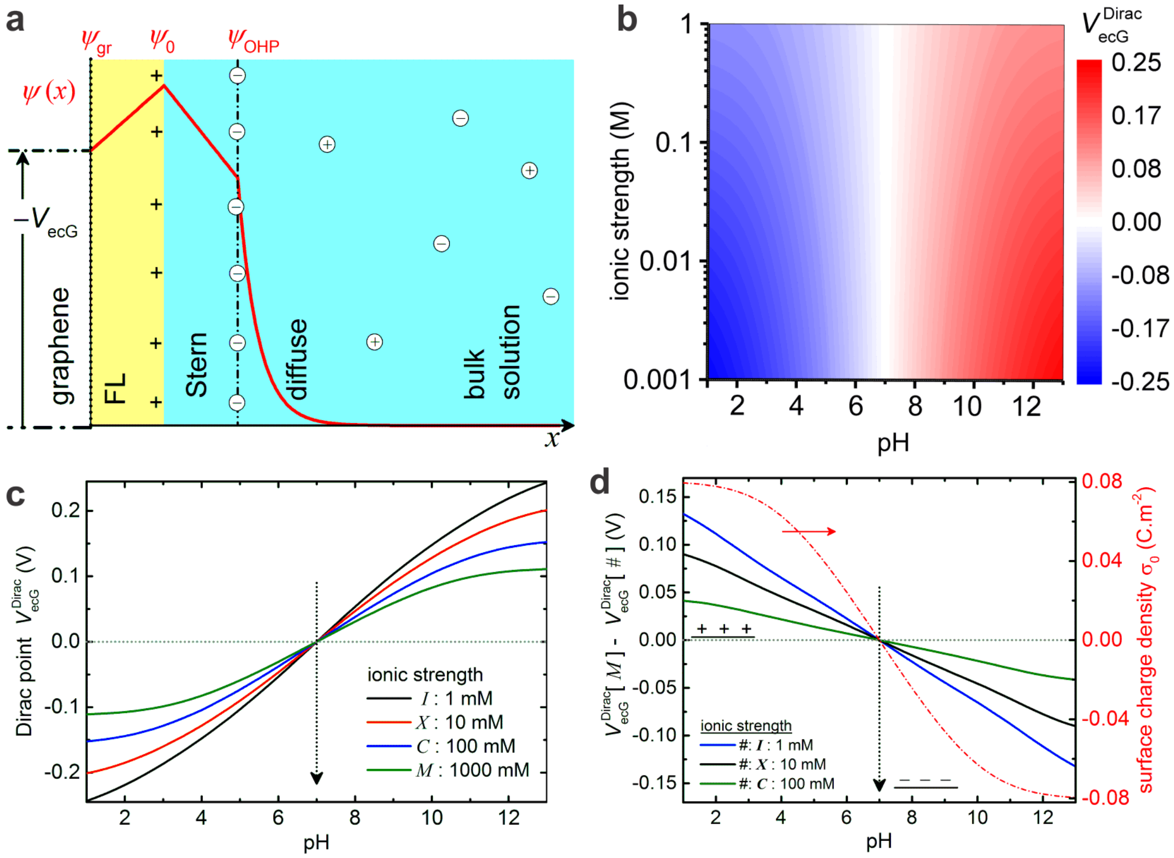

Figure S2. Calculate charge-potential relationships by including specific adsorption of ions (here anions) at the graphene-liquid interface. Same as in fig. 1 with the inclusion of specific adsorption of ions (indicated by the orange colored anions) at the inner Helmholtz plane (IHP). (a) Model of the graphene-liquid interface. (b) 2D-map of Dirac point gate voltage as a function of pH and ionic strength (IS). (c) Dirac point profile at 4 different IS values. (d) Difference Dirac curves *M-I, M-X, M-C* calculated from the profiles in (c). Superimposed is also the curve indicating the net surface charge density (on the right axis). Two important aspects are worth mentioning. The zero crossings of the difference curves shift to lower pH values (here only anions were included; there is no shift in the absence of specific adsorption as in fig. S1). The pH value of zero crossing of the *M-I* curve is very close to the value of the pI assumed in the model. The parameters are same as in fig. 1 (also see fig. S1) with the additional parameters of specific adsorption discussed in the text here.

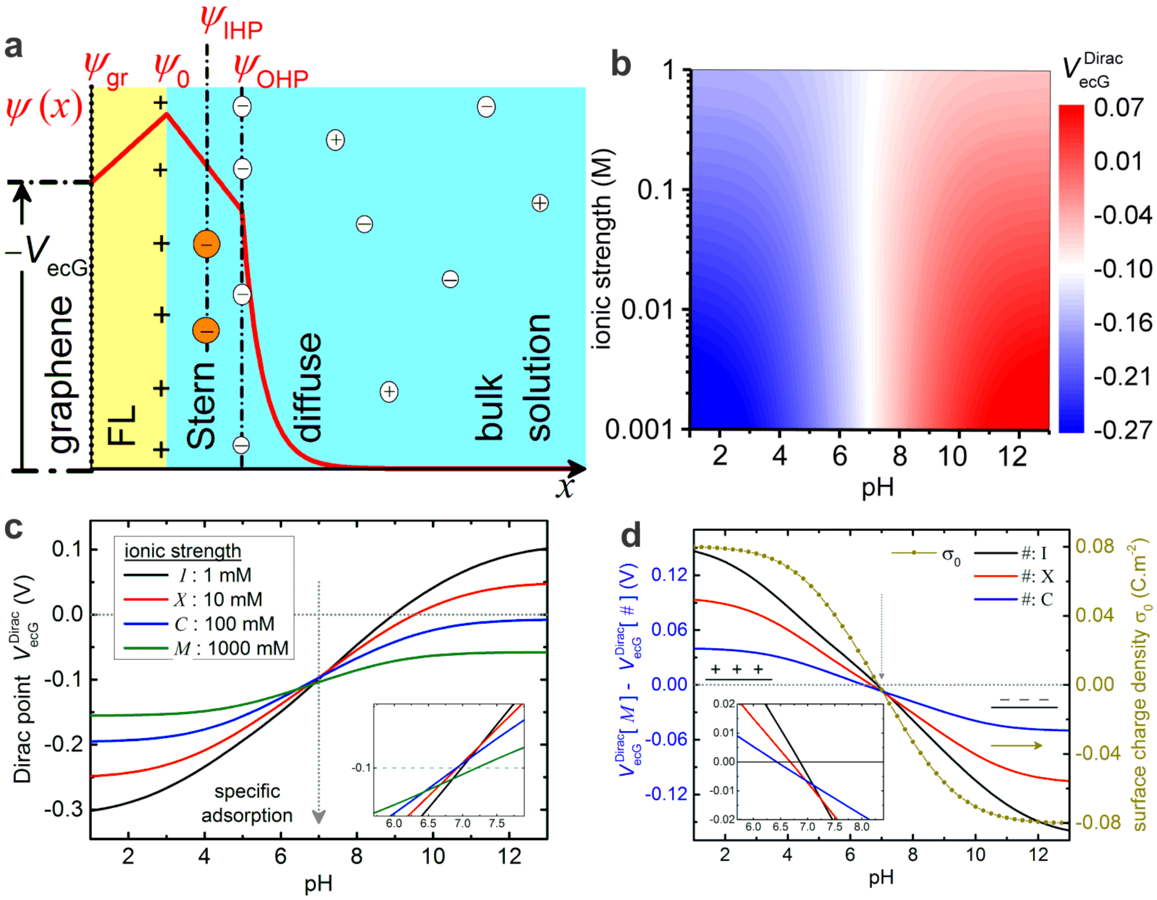

Figure S3. Effect of the nature of specific adsorption - cations vs. anions - on the electrical characteristics. (a,b) for anion adsorption, while (c,d) is for cation adorption. (a,c) Dirac point profiles at 4 different IS (b,d) Difference Dirac profiles for the cases of *M-I*, *M-X* and *M-C*. It is apparent that for the case of anions the shift of the zero crossing in (b) is to pH values lower than the pI, while for cations, the zero crossing shifts to higher pH values. In both cases, the zero crossing of the *M-I* curve occurs at a pH close to 7 indicating that it is a good estimate of the isoelectric point within +/- 0.2 pH units. The parameters for anion adsorption are: $K_{\mathcal{N}}^{\mathrm{gr}} = 50$ mol^{-1}L and $K_{\mathcal{C}}^{\mathrm{gr}} = K_{\mathcal{C}} = K_{\mathcal{N}} = 0$, while that for cations are: $K_{\mathcal{C}}^{\mathrm{gr}} = 50$ mol^{-1}L and $K_{\mathcal{N}}^{\mathrm{gr}} = K_{\mathcal{C}} = K_{\mathcal{N}} = 0$. The extent of adsorption represented by the parameter $\sigma_{\max}^{ii}$ is 7% for either case.

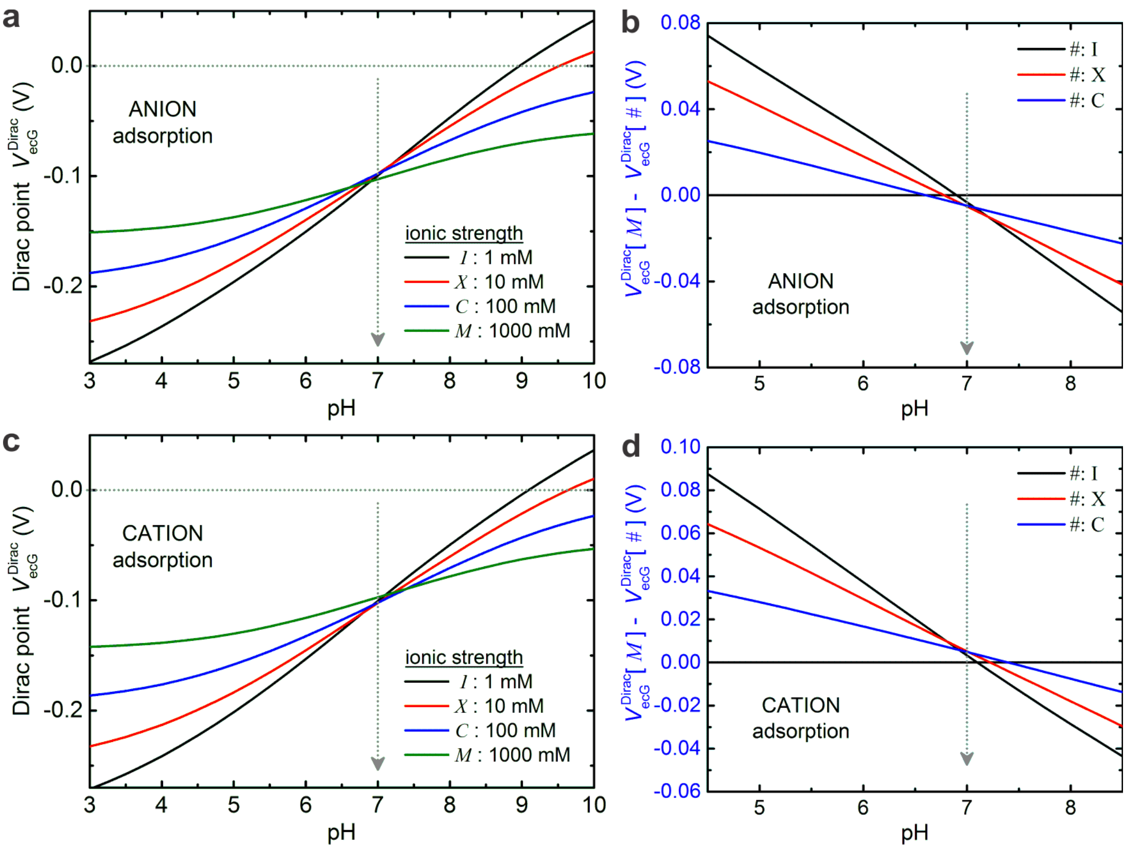

Figure S4. The isoelectric point of bare graphene. (Extended data set and fitted curves of figure 2). (a) Measured and (c) simulated Dirac point profiles of bare graphene as a function of pH for 4 different IS values. (b) Measured and (d) simulated difference Dirac curves for *M-I, M-X, M-C* signifying that the net charge is negative on the bare graphene surface. For the simulations a pI of 2 was assumed. See text in SI for further details of model parameters.

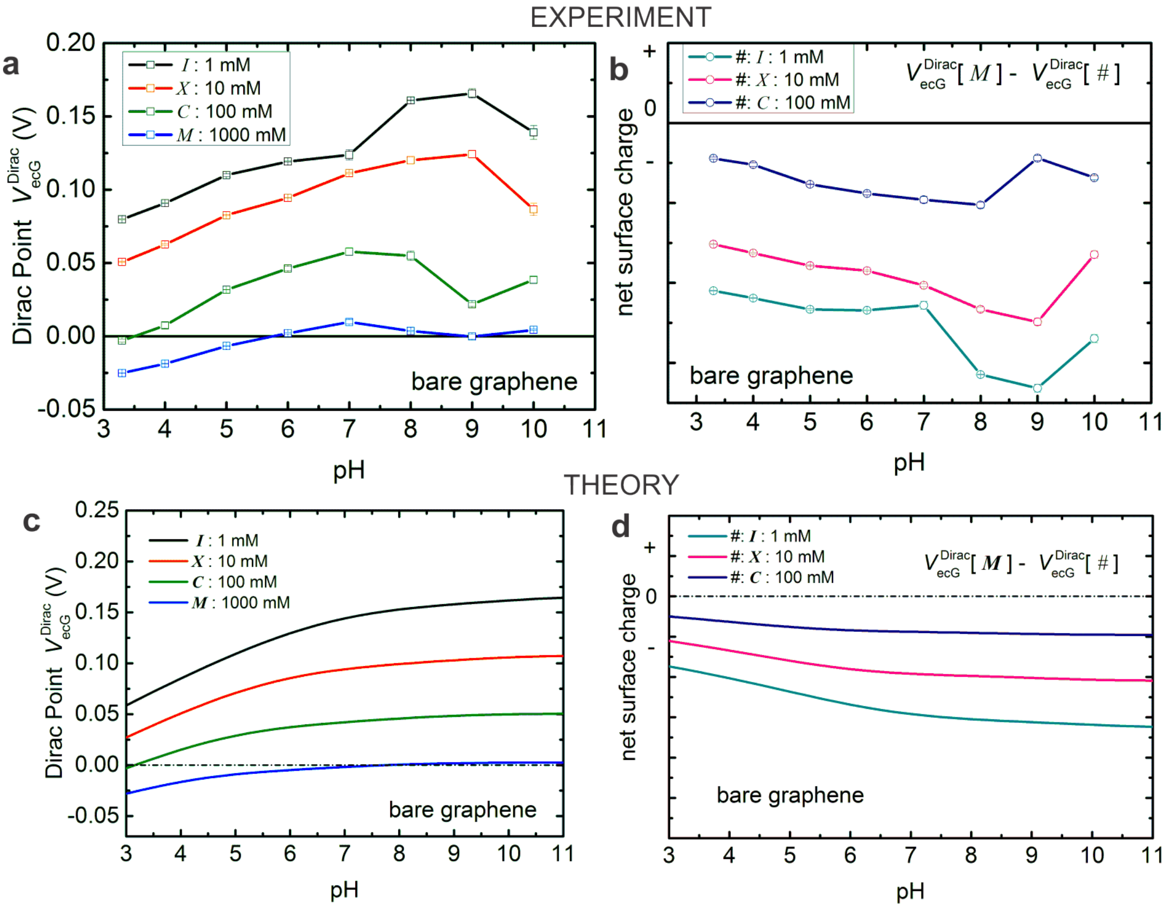

Figure S5. Typical Atomic Force Microscopy (AFM) images of a graphene device (a) before and (b) after electrochemical modification with *4*-aminobenzylamine (ABA). (c) Height profile along the white dashed line shown in (a). *x*,*y* – scale bar is 500 nm, *z*-scale bar is 50 nm. (d) Raman spectra obtained before and after modification with ABA, showing the absence of *D*-peak (~1300 cm^{-1}) signifying that the functionalities are attached non-covalently to graphene. Similar spectra were also obtained for the case of Gr/pANI.

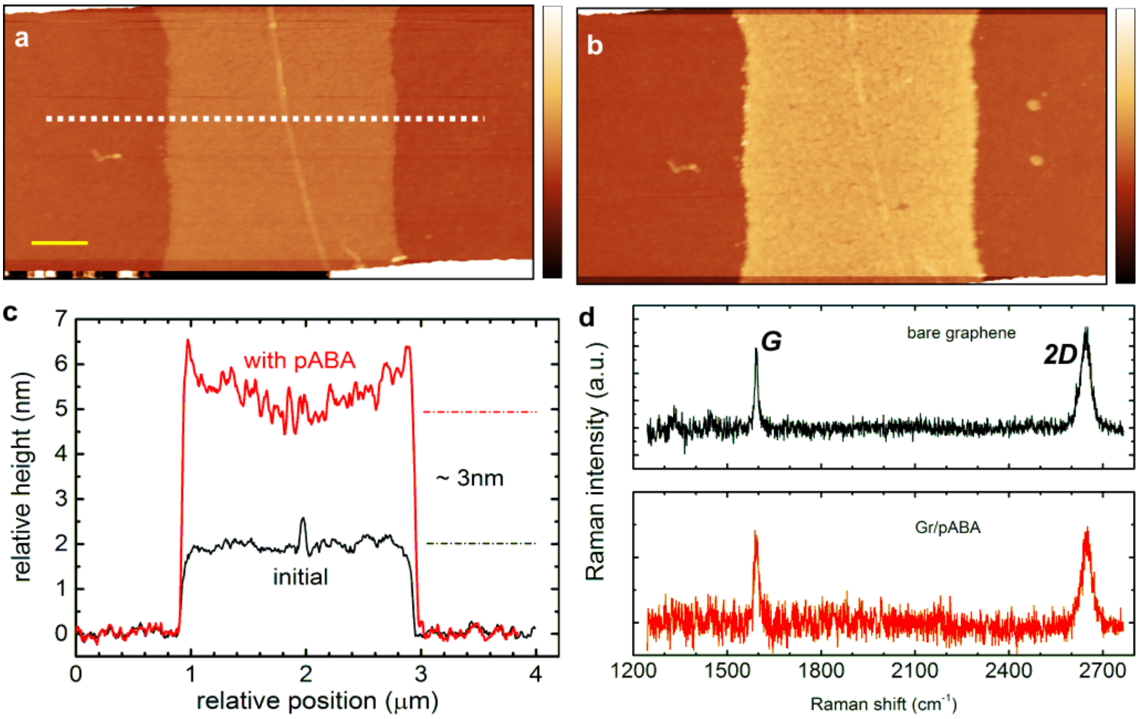

Figure S6. The isoelectric point of Gr/pABA (3 nm thick pABA coating on graphene). (Extended data set and fitted curves for the data in figure 4(a,b)). (a) Measured and (c) simulated Dirac point profiles of Gr/pABA as a function of pH for 4 different IS values. (b) Measured and (d) simulated difference Dirac curves for *M-I, M-X, M-C* representative of the net surface charge density. The shift in the zero crossings of the Dirac curves in the simulated curve in (d) is due to the inclusion of specific anion adsorption. See text in SI for further details of model parameters. The pI is found to be 5.98 and the pzc values are 5.98, 5.85, 5.65 and 5.6 at 1, 10, 100 and 1000 mM IS respectively.

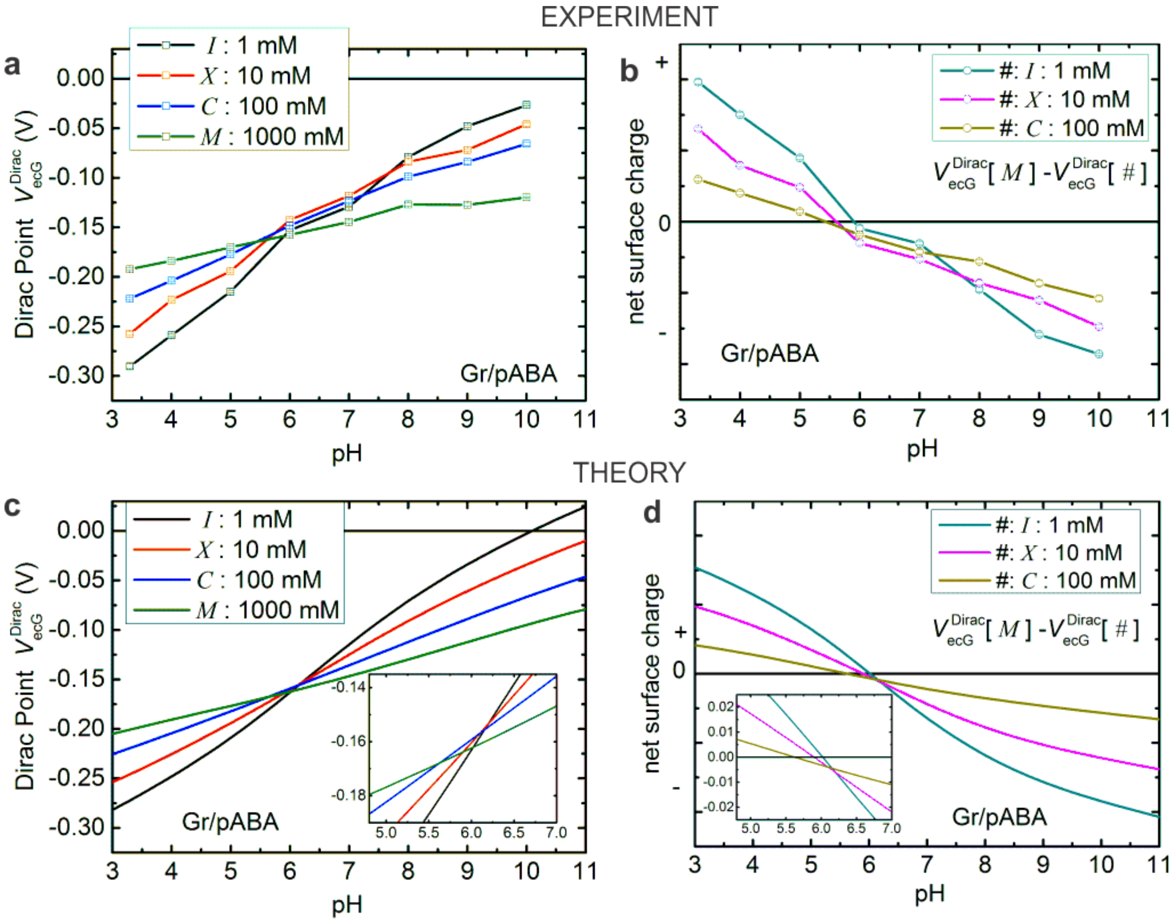

Figure S7. The isoelectric point of Gr/pANI (3 nm thick pANI coating on graphene). (Extended data set and fitted curves for the data in figure 4(c,d)). (a) Measured and (c) simulated Dirac point profiles of Gr/pANI as a function of pH for 4 different IS values. (b) Measured and (d) simulated difference Dirac curves for *M-I, M-X, M-C* representative of the net surface charge density. The shift in the zero crossings of the Dirac curves in the simulated curve in (d) is due to the inclusion of specific anion adsorption. See text in SI for further details of model parameters. The pI is found to be 3.85 and the pzc values are 3.82, 3.7, 3.45 and 3.38 at 1, 10, 100 and 1000 mM IS respectively.

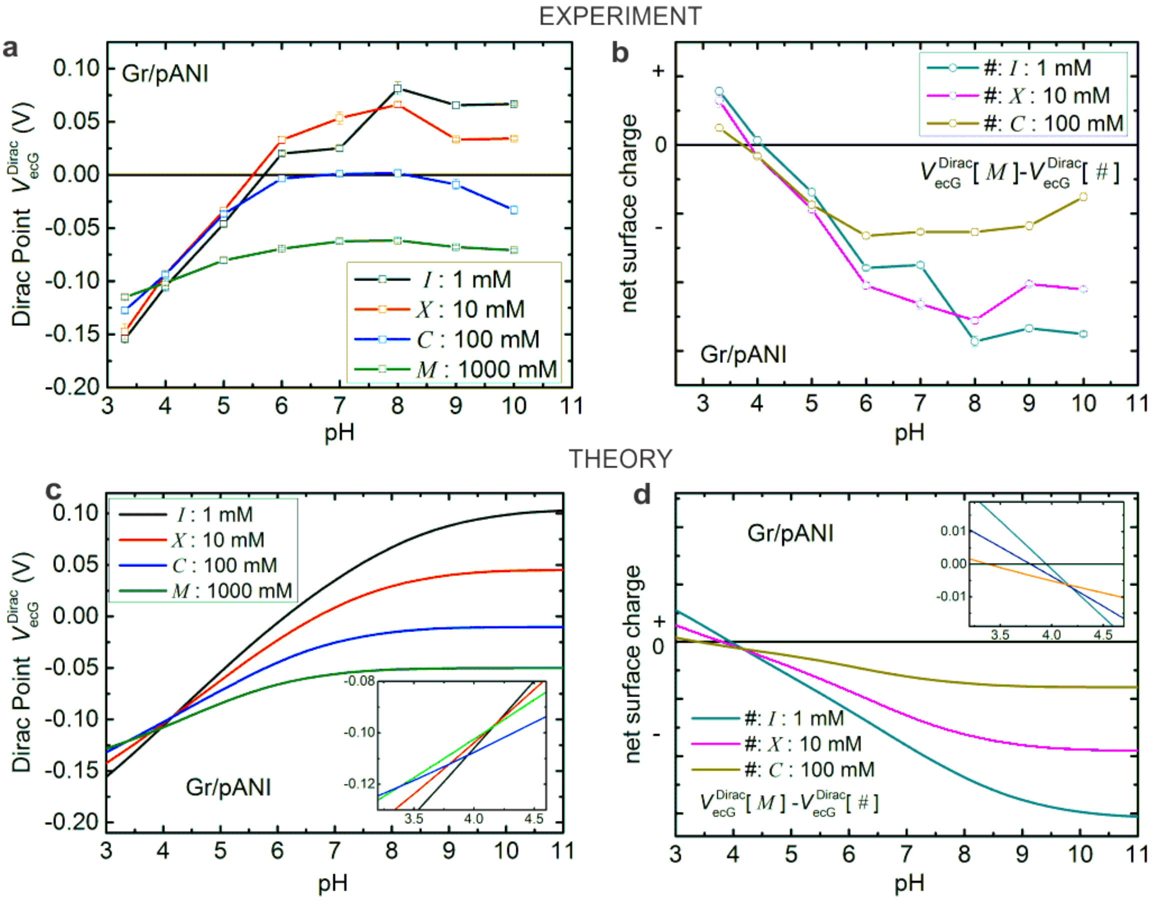

Figure S8. Comparison of the evolution of IEP (simulated) as a function of density of functionalities $\sigma_{\rm max}^{\rm FL}$ for two different types of functionalities each with a different pK_a. It is apparent that the maximum pI attainable on the functionalized graphene surface using a selected functional group is determined by its corresponding pK_a.

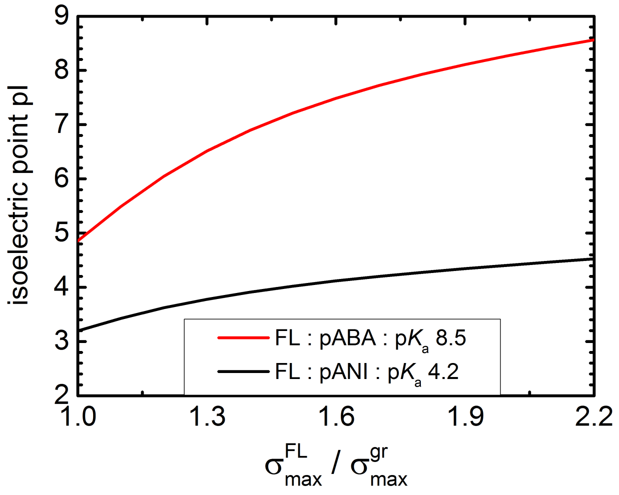

Figure S9. Effect of density of functionalities and substrate on the isoelectric point of functionalized graphene. (Extended data set for the data in fig. 5(a)). The pH value of zero crossings for the case of M-I, M-X and M-C are collected for devices fabricated on (a) Set 1 (dry oxide) and (b) Set 2 (wet oxide) substrates. The density of silanol groups is lower in the former in comparison to the latter type of substrates. It is apparent that the zero crossings *shift down in pH* in the order M-I , M-X and M-C for both sets of substrates signifying a predominant anion adsorption. However, for very large thickness we see an inversion in the trend of zero crossing. This indicates increased cation adsorption at high polymer thickness, presumably because of increased interaction of the ions with the functional groups of the pABA layer (in comparison to graphene).

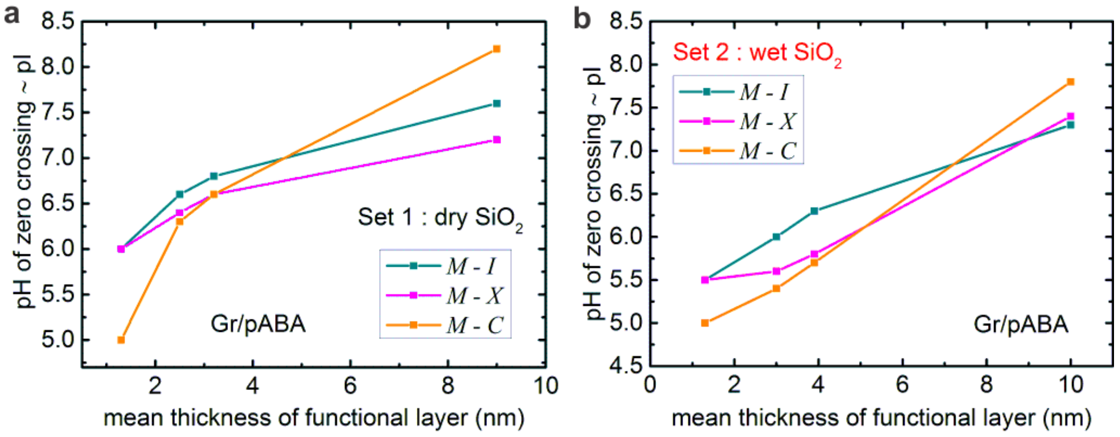

Figure S10. Tuning the isoelectric point of graphene. Raw data corresponding to figure 6 showing the measured Dirac profiles for the two ionic strength values (*I* and *M*) at the initial stage and after every ECM. The color coding is the same as in Fig. 6.

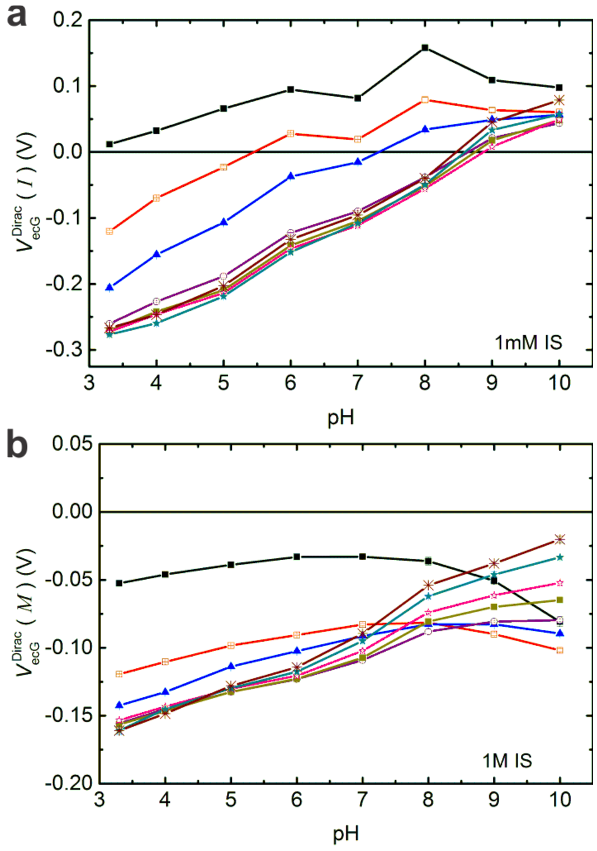

Supplementary References

1 Noh, J. S. & Schwarz, J. A. Estimation of the Point of Zero Charge of Simple Oxides by Mass Titration. *J Colloid Interf Sci* **130**, 157-164, (1989).

2 Yates, D. E., Levine, S. & Healy, T. W. Site-binding model of the electrical double layer at the oxide/water interface. *Journal of the Chemical Society, Faraday Transactions 1: Physical Chemistry in Condensed Phases* **70**, 1807-1818, (1974).

3 Heller, I. *et al.* Influence of Electrolyte Composition on Liquid-Gated Carbon Nanotube and Graphene Transistors. *J Am Chem Soc* **132**, 17149-17156, (2010).

4 Duval, J., Kleijn, J. M., Lyklema, J. & van Leeuwen, H. P. Double layers at amphifunctionally electrified interfaces in the presence of electrolytes containing specifically adsorbing ions. *J Electroanal Chem* **532**, 337-352, (2002).

5 Vanhal, R. E. G., Eijkel, J. C. T. & Bergveld, P. A Novel Description of Isfet Sensitivity with the Buffer Capacity and Double-Layer Capacitance as Key Parameters. *Sensor Actuat B-Chem* **24**, 201-205, (1995).

6 Duval, J., Lyklema, J., Kleijn, J. M. & van Leeuwen, H. P. Amphifunctionally electrified interfaces: Coupling of electronic and ionic surface-charging processes. *Langmuir* **17**, 7573-7581, (2001).

7 Back, J. H. & Shim, M. pH-dependent electron-transport properties of carbon nanotubes. *J Phys Chem B* **110**, 23736-23741, (2006).

8 Conway, E. S. in *Encyclopedia of Surface and Colloid Science* Vol. 3 (ed P. Somasundaram) 1908-1931 (CRC Press, Boca Raton, FL, 2006).

9 Ikada, Y. & Uchida, E. in *Encyclopedia of Surface and Colloid Science* Vol. 8 (ed P. Somasundaram) 6665-6675 (CRC Press, Boca Raton, FL, 2006).
